# Commercial Baby Foods: Nutrition, Marketing and Motivations for Use—A Narrative Review

**DOI:** 10.1111/mcn.70059

**Published:** 2025-07-02

**Authors:** Jasmine Brand‐Williamson, Alison Parrett, Victoria Sibson, Ada Lizbeth Garcia

**Affiliations:** ^1^ Human Nutrition, School of Medicine, Dentistry and Nursing, College of Medical, Veterinary and Life Sciences University of Glasgow Glasgow UK; ^2^ First Steps Nutrition Trust London UK

**Keywords:** commercial baby food, early years nutrition, food claims, food labelling marketing

## Abstract

A growing body of research on commercial baby foods (CBFs) has reported nutritional composition, marketing, and labelling concerns. We aimed to review and consolidate the evidence on CBFs marketed for children 0–36 months in the UK, Europe, Australia, and New Zealand to inform UK policy by highlighting key issues with the current retail offer. A systematic search conducted on PubMed and Web of Science on three topics: (1) Nutritional Composition, Flavour Profile and Texture, (2) Marketing and Labelling, and (3) Parental Choice and Preferences. Studies on CBFs were included if conducted in English in specified countries, age, and published between 2019 and 2024. Out of 3143 studies screened, 31 full papers were separated into three topics and reviewed. Topic 1: Out of all products sampled, 56% were puréed and 18% were snacks. The median sugar content per 100 g (IQR) were 10.4 g (1.0) in purées, 20.3 g (9.9) in snacks, and 14.7 g (14.4) in cereals. Nearly half of all products contained added or free sugars (*n* = 13 studies), and 62% when looking at snacks alone (*n* = 6 studies). Topic 2: Six out of 9 studies had ‘no added sugar’ claims, and eight studies reported finding claims related to health or nutrition. Topic 3: All studies reported that health/development/nutrition were motivations to purchase CBFs, and 75% mentioned ‘baby's enjoyment’, ‘convenience/time’, and ‘safety’. Purées and snacks dominate the CBF market and are often high in sugars. Marketing claims are misleading and exploit parents' fears to motivate use.

## Summary

- Commercial baby foods (CBF) are often nutritionally inadequate and/or high in sugar.
- Snack foods and squeeze pouches are readily available, often sweet, and lack textural variety.
- Marketing claims are often misleading and exploit parents/carers' fears to motivate use.
- Existing guidelines are not being utilised by baby food manufacturers.
- Policies, regulations, and advice on CBF must be strengthened to improve complementary feeding practices and reduce the prevalence of poor health outcomes.

## Graphical abstract

Commercial baby foods for infants aged 0–36 months are often nutritionally inappropriate and contain misleading marketing messages. Across 31 studies between 2019 and 2025 in the UK, Europe, Australia, and New Zealand, snacks and purées were most common and often high in sugars despite health and nutrition claims.

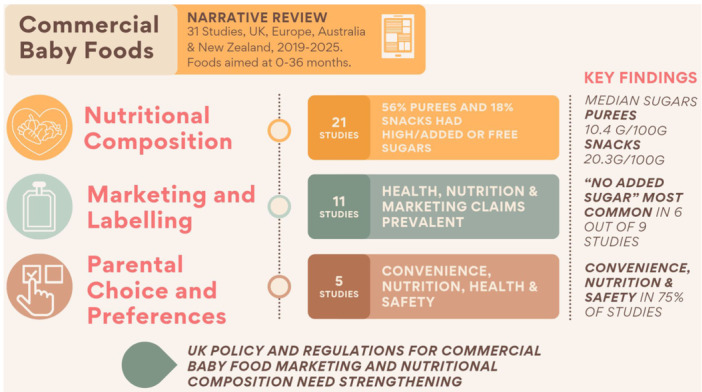

## 1 Introduction

Commercial baby foods (CBF) can contribute to poor diets, which could impact long‐term taste preferences in infants and young children (Mennella and Trabulsi 2012). Low‐quality diets for children under two are associated with an increased risk of obesity in later life and, in turn, type 2 diabetes, cardiovascular disease, and some types of cancer (Sahoo et al. 2015; Okubo et al. 2015). The complementary feeding period, from around 6 months of age to 24 months and beyond (WHO 2023), is crucial for optimal growth and development. An increasingly diverse, nutritious diet alongside breastmilk (or formula) is required for infants to introduce tastes and textures, and contribute to other developmental milestones, such as learning to chew and detecting hunger and satiety signals (Boswell et al. 2021). The UK Government's guidelines adhere to the World Health Organisation's (WHO) recommendations of exclusive breastfeeding for 6 months (WHO 2023). Infant formula is recommended for non‐breastfed or partially breastfed babies. After 6 months, the Scientific Advisory Committee on Nutrition (SACN) recommends that babies eat a diverse diet of home‐made foods, starting with less sweet vegetables (Table 1). From age 1–2 years, foods, flavours, and textures should continue to be diversified, and the Eatwell guide should largely apply by age 2 years (SACN 2023).

**TABLE 1:** International and UK public health recommendations and advice for complementary feeding.

	Complementary feeding guidelines	Commercial baby foods (CBF)	Sugar and salt	Energy and other nutrients	Marketing and labelling
WHO ‐ complementary feeding of infants and young children aged 6–23 months (WHO 2023)	– Start solids at 6 months, diverse diet, protein, fruit and veg daily – Pulses and nuts, frequently	Avoid unhealthy foods	– Avoid foods with sugar and salt – Avoid non‐sugar sweeteners	Exclude foods high in trans fats	Introduce policies for front‐of‐pack labelling
SACN – feeding in the first year of life and feeding young children aged 1–5 years (SACN 2018; SACN 2023)	– Start solids at around 6 months – Gradually introduce a wide range of foods, flavours, textures – Introduce vegetables on multiple occasions – Offer good sources of iron from 6 months	– Products high in sugars not recommended for between meals – Reduce sugars (especially in snacks) – CBFs not required to meet nutritional requirements	– Children over 1's sugar intakes should not exceed 5% of total energy intakes – Reduce free sugars – Salt should not be given	– Avoid excess protein – Avoid energy dense foods that are high in saturated fat, salt of free sugars	—
NHS – start for life advice (NHS 2024)	– Start solids at around 6 months – Variety in diet – Small amount of solid foods (start at once per day and build up) – Start with vegetables that are not sweet tasting, include bitter – Mashed or finger foods rather than puréed as soon as baby is ready	– Only home‐made recipes included – No mention of CBF usage – Avoid snack foods	– Avoid salt – Babies do not need sugar	– Main source of energy is breast milk or formula at the start of weaning	—

Despite their perceived convenience, CBFs can contribute to diets high in sugar and lacking textural variety. In 2018, SACN reported that 43% of five‐ to 7‐month‐olds in the UK were given CBFs (SACN 2018). A 2017 narrative review highlighted key concerns around CBF use, including inappropriate nutrient and energy contents, such as too much sugar, lack of food variety, sweet flavour profiles, and low meat/fish contents (Maslin and Venter 2017). Following this, Public Health England (PHE) highlighted the need for action in 2019 (Public Health England 2019). They found that young children were consuming diets that included too many snacks and were too high in salt and sugar. Additionally, they highlighted that appropriate homemade food is preferred as CBFs did not align with government advice on infant and child feeding or promote healthy diets. Their recommendations focused on improving CBF nutrient composition by reducing sugar, particularly in snacks. Further, they emphasised the need to limit misleading marketing practices, especially those contradicting public health recommendations for complementary feeding and to reduce the use of nutrition claims and ‘health halo’ statements (Public Health England 2019). Since then, the UK CBF market has continued to grow, with an estimated value of £774 million in 2023, nearly 70 million more than the previous year (Mintel 2024). One estimate suggests that the global market was worth USD 53.7 billion in 2024 and is likely to grow to 84.2 billion by 2033 (IMARC 2024).

There is a critical gap in public health advice for parents/carers in the UK regarding CBFs, as highlighted in Table 1. SACN clearly states that CBFs are not needed to meet nutritional requirements (SACN 2023). However, UK public health message gaps mean information may not reach parents/carers. For example, the National Health Service (NHS) official complementary feeding website does not provide guidance on CBF use for parents, despite it being a key resource for families. The NHS ought to provide recommendations on limiting CBF use as per SACN's suggestions. For food policy interventions to be effective, they should include policy actions for enabling environments, to overcome barriers, encourage healthy eating when purchasing, and focus on food‐system approaches (Hawkes et al. 2015). What's more, for food polices to have long‐lasting and equitable effects on health and obesity prevalence, they should prioritise enabling young children and infants to learn healthy food preferences, whilst also ensuring making healthy choices is easier and preferred (Hawkes et al. 2015). However, individual responsibility for health and nutrition is often prioritised over a supportive regulatory framework (Northcott et al. 2023). We see this in the UK, where the Office for Health Improvement and Disparities has avoided strengthening CBF regulations, and instead promised ‘Voluntary CBF and Drink Guidelines' with advice for manufacturers, which are yet to be published. Voluntary guidance is unlikely to effect change as observed in both environmental and food labelling issues (Aragòn‐Correa et al. 2020; Jones 2024).

Since PHE's 2019 review, CBFs are still not meeting the nutritional composition standards required to support healthy diets in children, and marketing and labelling practices remain misleading. puréed foods and snacks are readily available, as demonstrated by a 2021 cross‐sectional survey on 2634 products in 10 European countries, where 54% of products sampled in the UK were purées and nearly 20% were snacks (Hutchinson et al. 2021). Research suggests that snacks account for an increasingly high percentage of the UK baby food market. For example, a UK repeated cross‐sectional survey found that 21% of products sampled in 2019 were snacks compared to 10% in 2013 (Garcia et al. 2020). Similarly, in Iceland, snack foods accounted for 18% of products sampled in 2021, compared with 5% in 2016 (Thorisdottir et al. 2024). Snack foods are not needed for children under one, and breast milk or infant formula should be offered between food (WHO 2023b). By age one, up to two healthy snacks per day are recommended (WHO 2023b). Additionally, snacks are often high in sugars and have a melting texture and thus do not contribute to varied textural and flavour progression for infants as recommended by the SACN (Childs and Sibson 2023; SACN 2018). Highly processed snack foods are often referred to as ‘finger foods’ by food manufacturers and often carry marketing claims such as ‘encourages self‐feeding,’ which can suggest they are important to meet complementary feeding milestones (Garcia et al. 2024). puréed foods are often sold in pouches, high in free sugars, predominantly sweet in flavour, or consist of sweet ingredients such as fruit juice, syrups, dried fruits, or sweet‐tasting vegetables (Crawley and Westland 2019). A 2023 cross‐sectional survey in Australia found that 71% of 276 squeeze pouches contained fruit purée (Brunacci et al. 2023). Across all food categories, CBFs are often too high in sugars or favour sweet flavour profiles. In 2021, a cross‐sectional study found that 21%–58% of products contained added sugars, and 15% of savoury products in the UK contained processed fruit (Hutchinson et al. 2021). Additionally, CBFs are often high in sodium, fat, and water content, and low in energy density, iron, and protein sources.

Furthermore, marketing messages and on‐pack labels are frequently misleading. Pouches often lack proper guidance on both age appropriateness and safety. Babies sucking directly from pouches could hinder development of chewing skills and contribute to tooth decay, if high in free sugars, as this increases the time that the food spends in contact with their teeth (Crawley and Westland 2019). Many brands use health, safety, nutrition, or development claims to sell their products. For example, a 2023 cross‐sectional analysis from Portugal found that ‘no added sugar claims’ were present on nearly 70% of products, despite over 60% not meeting the nutritional standards set out in the WHO's Nutrient Profiling and Promotional Model (NPPM) (Santos et al. 2022). This is concerning given parents are more likely to purchase food with ‘no added sugar’ claims—around 14 times more likely in one parental choice survey (McCann et al. 2022). Further, a 2024 UK survey found 1003 emotional keywords on 341 products, with a median of three per product (Garcia et al. 2024). These messages can exploit parents' concerns and encourage the use of CBFs over healthier, home‐made equivalents, due to their perceived health, nutrition, and safety benefits. A 2022 survey, which interviewed 62 parents in the UK, determined that participants used CBF purées and snacks not only for convenience but also due to lack of confidence in their own food preparation skills, for safety, cost‐effectiveness, and because of sophisticated marketing (Isaacs et al. 2022).

High‐quality nutritional composition, marketing, and labelling guidelines for manufacturers of CBFs have been developed to improve the suitability of the market offer. The WHO's NPPM is a standardised tool to evaluate product suitability (WHO 2023). Research suggests that CBFs fall short of meeting the recommended requirements. For example, an Australian survey compared 250 products with the NPPM, only 43% of infant and 10% of toddler foods met all the nutritional requirements (Scully et al. 2023). In two recent studies, in the UK and Australia, no CBFs met the NPPM promotional requirements (Bozkir et al. 2025; Dunford et al. 2024).

Since Maslin's 2017 review and PHE's recommendations in 2019, it is necessary to consolidate newer emerging studies to assess progress following PHE's recommendations. The market is dynamic, and scrutiny of the nutritional composition and marketing strategies is necessary to ensure recommendations are in line with the current retail offer. Mandatory and strengthened regulations are needed to ensure that CBFs are appropriate for young children and to help promote healthy diets for young children in the UK. Given that one in eight UK toddlers are living with obesity before they start school (NHS England 2024) coupled with the UK government's inaction regarding CBF legislation and guidance, and the existing gaps in advice for parents and carers (Table 1), it is critical to provide an up‐to‐date review of the latest research. Therefore, this study aims to summarise, evaluate, and appraise the evidence on CBFs across three areas of concern: (1) Nutritional Composition, Flavour Profile, and Texture, (2) Marketing and Labelling, (3) Parental (or Carer) Choice and Preferences.

## 2 Methods

This literature review utilised a systematic search and a narrative perspective. The search focused on the UK due to the government's inaction on CBF guidelines and the continuing public health need to reduce obesity prevalence in young children. Australia, Europe, and New Zealand were also included due to the limited research conducted in the UK since the PHE report in 2019. These countries were deemed as having similar cultures, incomes, legislation, and market availability of CBFs. A keyword search was conducted on PubMed and Web of Science in late January 2025; articles were separated into topics in Microsoft Excel. Key search terminology included ‘CBF’, ‘packaged baby food’, ‘baby food pouches’, ‘nutrition’, ‘composition’, ‘promotion’, ‘advertising’, ‘marketing’, ‘homemade’ (Supporting Information: Table S1). A comprehensive list of research papers was divided into the topics: (1) Nutritional composition, Flavour Profile, and Texture, (2) Marketing and Labelling, and (3) Parental Choice and Preferences. As this review aims to synthesise evidence since the PHE review in 2019, papers before 2019 were not included. Further, topics 1 and 2 were developed from PHE's key recommendations regarding nutritional composition, flavour profile, texture, and marketing and labelling guidelines. Topic 1 had a wide scope, as many individual studies often had outcomes of interest relating to nutritional composition, texture, and flavour profiles combined. Topic 3 was added to understand motivations for use of CBFs, to better understand parents/carers' knowledge around CBF use, and any impacts from the gaps in advice. Studies that are related to multiple topics were included under each relevant topic. Publications were assessed if they met the following criteria:

- relevant to at least one of the above topics,
- published between January 2019 – January 2025 to capture all studies conducted following the 2019 PHE report,
- were conducted within the geographical locations of interest,
- were written in English,
- included products targeted at children aged 0–36 months (products are sometimes marketed at 4 months+ and parents/carers might introduce foods sooner than the 6 months+ guidelines recommend).

Studies were excluded if they were out of the geographical or age range, focused solely on metals, toxicity, allergens, or sustainability, or if CBFs were not mentioned in key outcomes of interest. Titles and abstracts were reviewed against eligibility criteria and separated into topics. If studies included countries outside of the country of interest, they were still included, but data from those countries were excluded. If there was any uncertainty about meeting the criteria, a second reviewer checked to eliminate bias. Reference lists of eligible studies were reviewed to capture a comprehensive list. Full papers were reviewed and the following data extracted where possible: (1) study design, (2) date, (3) population/setting, (4) age of participants, (5) outcomes of interest, (6) key findings or outcomes of study, and (7) statistical significance of outcomes. Outcomes of interest were included if they related to the topics of interest, such as, types and number of products sampled, nutritional information (amount of nutrient/100 g of products and/or % of products which met nutritional guidelines), prevalence and type of marketing claims and labelling information, and qualitative and quantitative values for motivations around choice for use. Data were then synthesised, and evidence tables were populated with information. Each paper was independently reviewed for quality assessment with an assessment checklist (BMJ 2016).

## 3 Results

**FIGURE 1:**
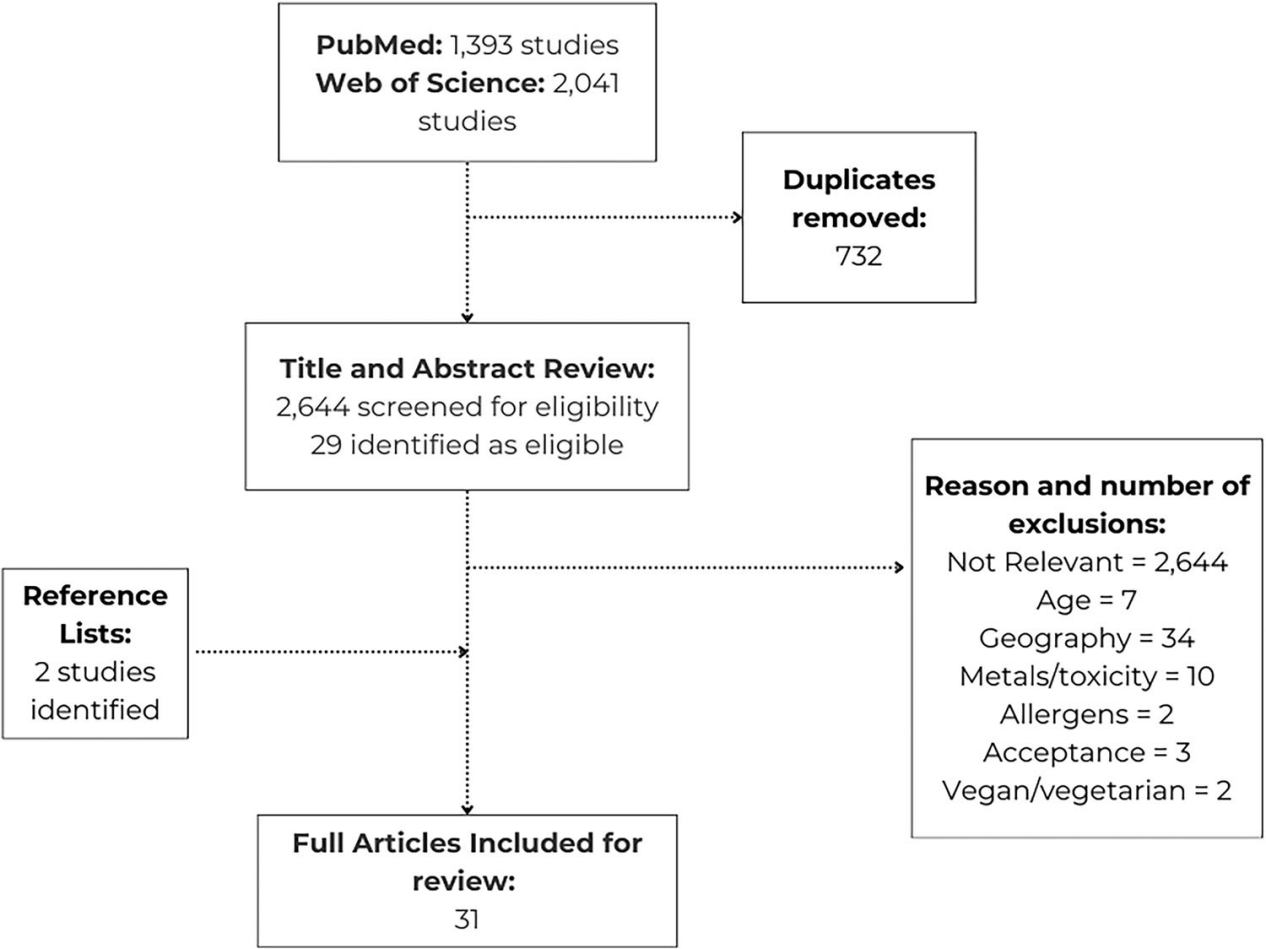
Study selection flow chart.

The search returned 3434 peer‐reviewed studies (Figure 1), with 30 eligible after title and abstract assessment. One further study was identified from a reference list. Out of 31 selected studies, 21 related to Topic 1, 11 studies to Topic 2, 7 of which were also in Topic 1, and five focused on Topic 3. Most (*n* = 27) scored 100% in the quality assessment (Supporting Information: Table S2). One study was longitudinal, and 30 cross‐sectional, of which 26 sampled products in‐store, online, or both, and aimed to sample every available product. Four studies were surveys/choice experiments, and one collected data from an online forum. Most focused on one country: ten on Australia, seven on the UK, four on Portugal, two on Germany, Spain and New Zealand, and one on Iceland, Italy and Malta. Others focused on two‐six countries, including Hungary, Norway, Portugal, Estonia, Slovenia, and Switzerland. One study included 27 European countries. Four studies surveyed 30–270 parents/carers, mostly women.

### 3.1 Topic 1: Nutritional Composition, Flavour Profile, and Texture

#### 3.1.1 General Characteristics

Studies sampled between 120 and 3427 products; those that sampled fewer were usually in one country or only sampled a particular food group (Table 2). On average, 64% of products sampled were puréed, soft, spoonable, or in pouches, and around a fifth were snack foods.

**TABLE 2:** Number of products sampled, country, and prevalence of squeeze pouches/purées and snacks/finger foods in products sampled by studies in topic 1.

Author, year	Products sampled (*n*)	Country	Squeeze pouches or purées (%)	Snacks/finger foods (%)
**UK**				
Bozkir et al. 2025	469	UK	47	16
Bassetti, E. et al. 2023	643	UK	69	20.4
Hutchinson, J. et al. 2021	768	UK	54	19
Garcia, A. L. et al. 2020	898	UK	62	21
**Europe**				
Thorisdottir, B. et al. 2024	473	Iceland	37	30
De Araújo, C. R. B. et al. 2023	123	Portugal	—	—
Santos, M. et al. 2022	191	Portugal	54	7.9
Garro‐Mellado, L. et al. 2022	120	Spain	—	—
Antignani, A. et al. 2022	285	Italy	46	44
Alexy, U. et al. 2022	1057	Germany	13.4	5.2
Hutchinson, J. et al. 2021	319	Denmark	74	9
''	241	Spain	54	5
''	414	Italy	53	11
''	243	Malta	58	18
''	99	Norway	81	16
''	125	Portugal	48	6
Grammatikaki, E. et al. 2021	3427	Europe	26.8	12
De Araújo, C. R. B. et al. 2021	431	Portugal	—	—
Theurich, M. A. et al. 2020	164 (only cereals)	Germany	—	—
Pace, L. et al. 2020	243	Malta	65	18
**Australia and New Zealand**				
Dunford, E. K. et al. 2024	309	Australia	58	18
Scully, M. et al. 2024	330	Australia	—	21
Scully, M. et al. 2023	250	Australia	71^c^	3^c^
''	''	''	9.6^d^	71^d^
Brunacci, K. A. et al. 2023	276	Australia	100^a^	—
Bassetti, E. et al. 2023	266	Australia	52.3	38.7
Katiforis, I. et al. 2021	266	New Zealand	63.9	22
McCann, J.R. et al. 2020	154	Australia	—	80
Padarath, S. et al. 2020	194	New Zealand	78	17
Moumin, N. A. et al. 2020	414	Australia	—	31.4
Median	276	—	56^b^	18

^a^ Only pouches sampled.

^b^ Study that included only pouches excluded from analysis.

^c^ Foods targeted at infants under 12 months.

^d^ Foods targeted at toddlers.

#### 3.1.2 Nutritional Composition

All topic 1 studies expressed concerns about sugar. Thirteen studies presented sugar content as medians or mean values per 100 g. The median sugar content per 100 g (IQR) was 10.4 g (1.1) in purées, 20.3 g (9) in snacks, and 15.4 g (14.4) in cereals (Table 3). Most purées had an average of around 10–11 g sugar per 100 g, and one New Zealand study, Katiforis et al. (2021), found a lower sugar content of 8.4 g/100 g (Table 3). For snacks/finger foods, two studies found finger foods had very high sugar content in Iceland (Thorisdottir et al. 2024) and Australia (Moumin et al. 2020), of 67.2 g and 59 g sugar per 100 g, respectively. Thirteen studies presented the percentage of products that contained added/free sugars, with a median of 43% overall (Table 4). Added sugars included syrups, honey, nectars, fruit juices, mono‐ and disaccharides. When categorised by food group, 22% of purées, 37% of cereals, and 62% of snacks had added/free sugars. Many savoury snacks still contained added/free sugars, around half in the UK, Denmark and Italy in Hutchinson et al.'s (2021) study (Table 4).

**TABLE 3:** The average sugar contents for squeeze pouches, snacks/finger foods, and cereals sampled and percentage of all products sampled containing sugar in topic 1 studies.

	Average sugar content in sampled products (g/100 g)		
Author, year	Purées	Snacks	Cereals
Bozkir, C. et al. 2025	—	*12.5* b	*16.2* b
Thorisdottir, B. et al. 2024	10^a^	67.2^a^	—
Brunacci, K. A. et al. 2023	9.8^b^	—	—
Santos, M. et al. 2022	11.0^a^	12.0^a^	27.2^a^
Garro‐Mellado, L. et al. 2022	—	—	25^b^
Antignani, A. et al. 2022	11.0^b^	22^b^	33^b^
Alexy, U. et al. 2022			
Infant (up to 1 year)	9.9^a^	7.5^a^	6.3^a^
Toddler (1–3)	10.8^a^	28.8^a^	8.5^a^
Katiforis, I. et al. 2021,	8.4^a^	22.3^a^	—
Grammatikaki, E. et al. 2021	—	16.1^b^	15.4^b^
McCann, J. R. et al. 2020			
Snacks	—	13.6^a^	—
Discretionary foods	—	22.7^a^	—
Theurich, M. A. et al. 2020	—	—	14^b^
Moumin, N. A. et al. 2020	10.9	59^a^	4.3^a^
Garcia, A. L. et al. 2020	—	20.3^a^	—
Average (IQR) (g/100 g)	10.4 (1.1)^a^	20.3 (9)^a^	14.7 (14.4)^a^

*Note:* sugar categories from NHS; dark orange = high sugars (> 22.5 g/100 g), orange = medium sugars (5–22.5 g/100 g) and green = low sugars (< 5 g/100 g) (Department of Health et al. 2016).

^a^ Median.

^b^ Mean.

**TABLE 4:** Products that contain added/free sugars or sweeteners for all products and by food type and country where available for topic 1 studies.

Author, year	Percentage of products that contain added/free sugars or sweeteners		
	All products		
Scully, M. et al. 2024	71		
Scully, M., et al. 2023			
Infants (under 12 months)	20		
Toddlers (1–3)	62		
Garro‐Mellado, L. et al. 2022	43.3		
Padarath, S. et al. 2020	34		
	Purée	Cereal	Snacks
Dunford, E. K. et al. 2024	90	62	51
Brunacci, K. A. et al. 2023	20	—	—
Bassetti et al. 2023			
Australia	22	18	51
UK	5	39	68
Santos, M. et al. 2022	4.8	33	55.6
Antignani, A. et al. 2022	44	—	91
Hutchinson, J. et al. 2021	32	8	56
UK	35	18	97^a^
			43^b^
Denmark	12	33	100^a^
			50^b^
Spain	61	62	100^a^
Italy	35	62	100^a^
			50^b^
Malta	11	45	78^a^
			20^b^
Norway	18	29	73^a^
			40^b^
Portugal	36	44	100^a^
Grammatikaki, E. et al. 2021	—	35	39
Theurich, M. A. et al. 2020	—	—	100
Pace, L. et al. 2020	11	45	77^a^
			20^b^
Median categories	22	37	62
Median ALL products and categories combined	43		

^a^ Sweet.

^b^ Savoury.

**TABLE 5:** Types of on‐pack marketing and labelling claims in topic 3 studies (marketing and labelling).

Author, year, location	Themes of marketing claims				
	Health (%)	Nutrition (%)	No added sugar (%)	Emotional/idealisation (%)	Quality/premiumisation (%)
Garcia, A. L. et al. 2024, UK	—	—	—	Emotional key words (60)	Quality (12)
Brunacci, K. A. et al. 2023, Australia	Digestive health (2) Bone health (10), Immune system (1), Development (8)	No added salt (44), No artificial colours (92), No artificial flavours (95), No preservatives (90), Probiotic (7)	59	Good parenting (26)	Premiumisation (41), organic (40)
Santos, M. et al. 2022, Portugal	91	No added salt (27.2), No preservatives (29.8), Gluten free (33.5)	69.6	—	—
McCann, J. R. 2022, Australia	26	73	—	—	
Garcia, A. L. et al. 2022, UK	1	85	58	Ideal feeding claims (30)	63
Simmonds, L. et al. 2021, Australia	17	39.4	55	—	—
De Araújo, C. R. B. et al. 2021 (Processed Foods), Portugal	Growth, Allergy, Cognitive, Immune (7.1), Other, 93	Fortified with vitamins (7.1), Gluten free (50), No added salt (53.6)	14.3	—	—
De Araújo, C. R. B. et al. 2021 (UPFs), Portugal	Cognitive, (3.6), Other (88.6)	Fortified with vitamins (49.7), Gluten‐free (25.8), Fortified with minerals (56.9)	29.4	—	—
McCann, J. R. 2020, Australia	Health‐related ingredient (94), General health (16)	61	—	—	Organic (42)

Other nutrients were not consistently reported; however, some studies compared the nutritional composition to specific guidelines. For example, the WHO NPPM was used as a comparison tool with guidelines for energy density, sodium, sugar, added free sugar or sweeteners, protein, fat, and fruit content (WHO 2023). One study in the UK (Bozkir et al. 2025) found that most products met the criteria for energy content (75%) and protein (94%). However, less than half (45%) of products met all recommendations (Bozkir et al. 2025). Four studies in Australia (Pace et al. 2020; Scully et al. 2023; Scully et al. 2024; Dunford et al. 2024) found that between 22% and 36% of products met the WHO NPPM requirements, as many failed on sugar. Three papers reported on iron content; Theurich et al. (2020) found that 26% of cereal products in Germany contained iron, and Moumin et al. (2020) found that most products were poor sources of iron in Australia.

#### 3.1.3 Texture and Flavour Profile

Smooth products accounted for a higher percentage of products in Denmark (74%) and Norway (81%) compared with other countries (Table 2). Around 50%–70% of products were classified as smooth across all regions (Table 2). Alexy et al.'s (2022) study found that 13% of products were smooth in Germany; however, only the texture for main meals was reported (Table 2). The variation is likely due to differences in the categorisation of foods across studies. Few studies reported on flavour profile; however, Thorisdottir et al. (2024) found that the percentage of food classified as sweet increased from 65% to 77% between 2016 and 2021 in Iceland. In Australia, Katiforis et al. (2021) found that 66% of products were classified as sweet. Garcia et al. (2020) found that many savoury products contained 50% or more sweet root vegetables in the UK, and that 29% contained fruit juice. Similarly, Brunacci et al. (2023) found bitter vegetables in less than 10% of products in Australia.

### 3.2 Topic 2: Marketing and Labelling

Most studies focused on the presence of on‐pack claims. Six out of 9 studies had ‘no added sugar’ claims, with nearly 70% of products using the claim in Portugal (Santos et al. 2022), and 60% in both the UK (Garcia et al. 2022) and Australia (Brunacci et al. 2023). Eight studies reported finding claims related to health, such as growth or immunity claims, or nutrition, such as ‘calcium for strong bones’ (Simmonds et al. 2021). Emotional keywords, as defined by an adapted ‘hierarchical consumer emotions model’, were identified in 30% of all products in Garcia's study in 2024 (Garcia et al. 2024).

### 3.3 Topic 3: Parental Choice and Preferences

All four studies mentioned health/development/nutrition as an incentive for purchasing, and 75% mentioned ‘baby's enjoyment’, ‘convenience/time’, and ‘safety’. In the UK, Hollinrake et al. (2024) found that 95% of participants mentioned ‘convenience/time’, 79% ‘baby's enjoyment’, 75% ‘safety’, and 60% ‘health/development/nutrition’. A UK qualitative study found that parents mentioned trusting certain brands and fears around cooking themselves (Isaacs et al. 2022). In Australia, McCann et al. (McCann) found that parents were 13.7 times more likely to choose products with ‘no added sugar’ or ‘salt’ claims. Similarly, in Australia, Dixon et al.'s 2024 survey found parents were more likely to choose unhealthy food products when they contained a ‘free from’ ingredient claim (Table 5).

## 4 Discussion

Our review found that CBFs remain nutritionally inadequate, often high in sugars and continue to use inappropriate marketing which is against the WHO NPPM guidelines. CBFs are used by parents largely driven by convenience, perceived safety, and marketing influences. These findings reinforce the need for strengthened, mandatory UK CBF regulations with independent monitoring and enforcement. The NHS complementary feeding advice must be updated to include explicit guidance on CBFs.

The findings from this review show there has been minimal progress towards improving CBF quality, and on‐pack marketing claims. Foods marketed for children aged 0‐36 months are still nutritionally inadequate, high in sugars, favour sweet flavour profiles, and lack textural variety. Further, snacks for babies and toddlers are increasing in availability, an analysis of the baby food market found that between 2019 and 2023, ‘baby snack’ product launches grew from 10% to 15% of the overall market worldwide (Innovia 2023). Similar findings were observed in Garcia's and Thorisdottir's surveys in the UK and Iceland (Garcia et al. 2020; Thorisdottir et al. 2024). Marketing and promotional claims remain pervasive and target parents' and carers' fears to gain sales (Isaacs et al. 2022). These concerns mirror those discussed in Maslin's review in 2017 and show little progress towards recommendations outlined by PHE in 2019. This is reflected by the findings that in Bozkir et al.'s 2025 UK cross‐sectional study, only 45% of products would meet the WHO's NPPM guidelines. These recent findings echo those found in 2020 and 2024 by Pace et al. (Malta) and Scully et al. (Australia), where just 36% and 28% of products would meet the WHO's NPPM guidelines, respectively. As such, CBFs do not support optimum healthy diets for children during the complementary feeding period and up to 36 months of age, as they are regularly not meeting the standards, as recommended by the WHO.

This review reinforces that squeeze pouches/purées lacking in textural variety are commonly available, accounting for the majority of the market in most studies, and as high as 70%–80% in at least one study in all regions (Bassetti et al. 2023; Hutchinson et al. 2021; Scully et al. 2023; Padarath et al. 2020). Sucking directly from pouches does not encourage the development of chewing skills as food must be smooth to fit through the spout (Crawley and Westland 2017). Food acceptance can also be impacted by texture—one French longitudinal study found higher acceptance for varied textures when infants ate with their fingers and consumed CBFs (such as purées) less often (Tournier et al. 2021). Children may struggle to smell and taste food, and to control the portion sizes they consume from pouches (Crawley and Westland 2017). Most purées predominantly contain fruit, and few purées included dark green vegetables (Bassetti et al. 2023), and even when they do contain vegetables, they are often masked by fruit and unlikely to contribute to acceptance of new flavours (Bakke et al. 2020). Similarly, pouches are especially likely to contain high levels of free sugars, and some products had sugar levels as high as 76.2 g/100 g. Additionally, fruits are often acidic which in combination with sugar has a role to play in the mouth cavity in regards dental health, however, there is no research yet to understand how this could affect oral microbiota. A 2022 systematic review and meta‐analysis found that although children were more likely to have dental caries with high Ultra‐proccessed food (UPF) consumption, evidence was stronger when looking at associations between high sugar and starch consumption with caries (Cascaes et al. 2022). More high‐quality research is needed into the associations between the degree of processing, nutritional composition and dental caries. Information on free sugar levels is not usually available as it is not a labelling requirement, and as such reported sugars (from added/total sugars) can be lower than the real amount. One 2014 publication found that laboratory analysed sugar content was nearly four times higher than the reported amount in some purées in the US, but more research is required in this area (Clifford et al. 2014). This is especially concerning given that when Action on Sugar (2022) asked CBF pouch manufacturers in the UK for information on free sugars, just two out of 10 were able to report on free sugar amounts, and others reported containing no free sugars despite products containing fruit purées—which are classified as free sugars. Therefore, the rise in the availability of pouches and the high prevalence of puréed foods remain a problem.

Similarly, snack food availability continues to rise, as noted in this review and previous studies (Innovia 2023; Thorisdottir et al. 2024; Garcia et al. 2020). The NHS states that babies under 12 months do not need snacks, and the WHO recommends that commercially available snacks high in salt, sugar, and fat should not be consumed by young children (NHS England 2024; WHO 2023a). Despite this, snack foods (excluding confectionary) are given to 74% eight‐12‐month‐old children in Scotland daily, and 34% of UK babies aged four‐6 months are given baby snacks (Scottish Government 2017; Public Health England 2019). Snacks and finger‐foods are especially likely to be high in sugars per 100 g (Scully et al. 2023 and Scully et al. 2024), although may be offered in smaller portion sizes of 5–50 g for snacks compared with 120–150 g for puréed products (Garcia et al. 2013). With regards to texture, the findings that nearly half of savoury snacks were ‘extruded puffs’ from Moumin's 2020 study are concerning as these are low in fibre and can lead to reduced satiety as they can be eaten faster (Moumin et al. 2020). Our findings of high prevalence of ‘no added sugar claims’ reflect an Action on Sugar report, which found they were present on 75% of 97 products sampled (2022). Action on Sugar found that 84% of 1000 survey participants buy ready‐made snacks for their children aged one‐3 years, and 59% would be motivated to buy them if they had a ‘no added sugar claim’ (Action on Sugar 2019), reflecting findings in this review (McCann et al. 2022). It is concerning that products with ‘no added sugar’ claims were high in free sugars or contained sugar‐contributing ingredients (Grammatikaki et al. 2021). These nutritional claims can act as health halos causing parents to believe CBFs are more nutritious than in reality (Action on Sugar 2019). A 2022 randomised control trial (RCT) in France found that reformulation by reducing sugar in CBFs did not alter the acceptance (Sanchez‐Siles et al. 2020), similar to a RCT which found that pouches with reduced sugar and less processed ingredients were highly accepted by babies in Spain (Klerks et al. 2022). The baby's enjoyment of many CBFs is unlikely to be altered by sugar reduction.

This review and previous studies demonstrate that sugar levels in CBFs are concerning (Crawley and Westland 2017; WHO 2019). The reported average sugar contents of between 10.8 and 20.3 g of sugar per 100 g of purées, cereals and snacks is high. Similarly, the high prevalence of added and free sugars (particularly fruit juice) in nearly half of the products sampled and 70% in snack foods goes against public health guidance. This is especially true given that babies should not consume sugar and infants over 1 need to limit their free sugar intakes to reduce the risk of dental caries and a long‐lasting sweet taste preference (Mura Paroche et al. 2017). Recent research that found limiting sugar intakes before age 2 can lead to a reduced risk of hypertension and diabetes (Conway et al. 2024).

Marketing claims are linked to the motivations for CBF use, as demonstrated by McCann et al.'s (2022) choice experiment, which found that parents/carers are more motivated to purchase CBFs if they have a ‘no added sugar’ claim. Marketing and promotional on‐pack claims are still extensive; convenience, health, safety, baby's enjoyment and trusting of certain brands have all been mentioned (Isaacs et al. 2022; Hollinrake 2024; McCann et al. 2022; Rowan et al. 2022). Lack of on‐pack labelling regulations mean parents/carers' fears can be exploited to motivate CBF use. The claims observed are misleading, pervasive, and often do not reflect the nutrient contents (Carstairs et al. 2016). Bassetti et al. (2023) found products with ‘no added sugar’ claim contained sugar‐contributing ingredients or free sugars. Further, emotional claims such as ‘smile from the inside’ and ‘happy tummies’ are common (Garcia et al. 2024 and Simmonds et al. 2021). These messages motivate CBF use due to their perceived health or wellbeing benefits.

Both PHE and SACN state that homemade foods are preferable to CBFs (Public Health England 2019; SACN 2023). Originally, the scope of this study included a comparison between CBFs and homemade baby foods, yet only one paper addressed this topic, which calls for more research in this area. Bernal et al. (2021) et all found that fibre was significantly higher for homemade foods compared to CBF (infants, 2.6 g v 0.8 g, *p* < 0.001, young children, 2.0 g v 0.8 g, *p* < 0.001) while median energy was 43% higher for commercial foods vs homemade. It found that homemade foods had more types of vegetables albeit this difference was small (3.3 different types of vegetables for homemade vs 3.7 in commercial). Maslin's 2017 narrative review called for more research comparing CBFs and home‐made equivalents, and this present review reflects that this gap remains. First Steps Nutrition Trust (FSNT) compared the taste, texture, and price of homemade and CBFs (2017). Reflecting previous findings (Carstairs et al. 2016), CBFs were generally more expensive even when the homemade equivalents were made with higher proportions of expensive ingredients. For example, a homemade ‘Sunday chicken dinner’ was 99p cheaper per portion than CBF and contained 25% rather than 13% chicken per portion (Crawley and Westland 2017). Adults who tested CBFs reported that they were mostly bland despite being very sweet (Crawley and Westland 2017).

Key strengths of this review include its wide scope, covering the nutritional composition, marketing, and labelling of CBFs and the motivations for their use. It provides a recent, comprehensive review that captures market changes since PHE's 2019 report. A limitation is the synthesis of data from studies with varying methodologies and geographies. Differences in how studies reported nutritional content (mean, median, percentages) and the omission of portion sizes made direct comparisons challenging. The wide geographical scope of this review allows us to observe trends across countries. However, due to varying cultural approaches to diet, and differences in food availability across the UK, Europe, Australia, and New Zealand, we cannot be certain of their relevance for the UK's policy environment. The US, Canada, and other English‐speaking countries were excluded due to differences in their cultural and legislative landscapes and is a limitation. Although this review includes international data, variations in local dietary practices, regulatory environments, and cultural factors might slightly affect applicability directly to the UK. Nonetheless, trends observed internationally provide valuable insights relevant to UK policy development.

Further, most studies reviewed were cross‐sectional surveys, making it hard to draw strong conclusions. RCTs are not feasible in this age group due to logistical and ethical considerations (Henschel et al. 2010). Another limitation is the omission of reporting on the degree of processing of products, due to this being an emerging research area where there appears to be associations between UPF consumption and children's health (Childs and Sibson 2023). However, only three studies looked at the degree of processing, so future studies could incorporate this information. Future research could include a review of CBF age guidelines, expanding the scope to drinks, the inclusion of studies covering toxicity from CBF packaging (Rasic Misic et al. 2022), sustainability and environmental impact, allergens in CBFs (Netting et al. 2020) and concerns with energy density.

## 5 Conclusion

This narrative review further highlights that despite PHE's advice to the Government in 2019 on actions to improve the nutritional composition, marketing, and labelling of CBFs, the same recurring issues remain. High‐quality guidelines already exist, such as the WHO's NPPM, but these are not being utilised. Future research should compare home‐made and CBFs and examine potential long‐term associations between CBF use and poor health outcomes such as obesity and non‐communicable diseases, as well as including in‐depth textural analyses. Study designs should include longitudinal studies to evaluate changes in CBF quality over time.

The wider policy recommendations from this narrative review are to:

1. Strengthen regulations in the UK and ensure that they are mandatory.
2. Ensure independent monitoring and enforcement of regulations.
3. Clarify NHS complementary feeding advice to include explicit guidance on CBFs.

These steps are necessary to enable parents/carers to make informed decisions about what foods to choose when feeding their infants and young children, in line with public health recommendations. Mandatory guidelines are essential as voluntary ones historically are ineffective. Improved diets in the infants would help to reduce the prevalence of obesity and non‐communicable diseases, therefore allowing children to grow up into healthy adults.

## Author Contributions

Jasmine Brand‐Williamson, Victoria Sibson, Ada Lizbeth Garcia, and Alison Parrett conceptualised the research study. Jasmine Brand‐Williamson designed the research study and performed the research. Alison Parrett and Ada Lizbeth Garcia supervised the research and performed secondary review during the literature search stage. Victoria Sibson contributed essential knowledge of policy and performed a secondary review of the grey literature reference list. Jasmine Brand‐Williamson wrote the first paper draft. All authors reviewed the final manuscript.

## Ethics Statement

The authors have nothing to report.

## Conflicts of Interest

The authors declare no conflicts of interest.

## Supporting information

Supplementary Material 1.

Supplementary Material 2.

## Data Availability

The data that support the findings of this study are available from the corresponding author upon reasonable request.
