# Supplementary material for: Commercial Baby Foods: Nutrition, Marketing and Motivations for Use—A Narrative Review
Source: Matern Child Nutr. 2025 Jul 2;21(4):e70059. doi: 10.1111/mcn.70059 (PMC12454211; doi:10.1111/mcn.70059)
Supplement: Supplementary file 1 — Supplementary Material 1. [file MCN-21-e70059-s001.docx]

**Supplementary Table 1 - PubMed and Web of Science Search Criteria and Results**

| **PubMed Search Criteria** | **Timeframe** | **Search Results** |
| --- | --- | --- |
| (commercial baby food) OR (commercial infant food) OR (commercial toddler food) OR (commercial preschool food) OR (packaged baby food) OR (packaged infant food) OR (packaged toddler food) OR (packaged preschool food) OR (baby food pouches) OR (infant food pouches) OR (toddler food pouches) OR (preschool food pouches) AND (nutrition OR composition OR quality OR flavour OR promotion OR advertising OR marketing OR market OR message OR messaging OR labelling OR on-pack OR homemade OR home-cooked) | January 2019 – January 2025 | 1,393 |
| **Web of Science Search Criteria** |  |  |
| (commercial baby food) OR (commercial infant food) OR (commercial toddler food) OR (commercial preschool food) OR (packaged baby food) OR (packaged infant food) OR (packaged toddler food) OR (packaged preschool food) OR (baby food pouches) OR (infant food pouches) OR (toddler food pouches) OR (preschool food pouches) AND (nutrition OR composition OR quality OR flavour OR promotion OR advertising OR marketing OR market OR message OR messaging OR labelling OR on-pack OR homemade OR home-cooked) | January 2019 – January 2025 | 2,041 |

Supplementary Table 1 shows the key words used in the peer-reviewed literature search. Search was carried out January 2025
