# Supplementary material for: Commercial Baby Foods: Nutrition, Marketing and Motivations for Use—A Narrative Review
Source: Matern Child Nutr. 2025 Jul 2;21(4):e70059. doi: 10.1111/mcn.70059 (PMC12454211; doi:10.1111/mcn.70059)
Supplement: Supplementary file 2 — Supplementary Material 2. [file MCN-21-e70059-s002.docx]

**Supplementary Table 2 – Quality Assessment of Peer-Reviewed Literature** †

| *Study Author, Year* | *Score out of 20* | *Number of N/A answers* | *Percentage compliance (%)* |
| --- | --- | --- | --- |
| Bozkir et al, 2025 | 15 | 5 | 100 |
| Dunford , E. K. et al, 2024 | 15 | 5 | 100 |
| Thorisdottir, B. et al , 2024 | 15 | 5 | 100 |
| Scully, M. et al, 2024a | 16 | 4 | 100 |
| Scully, M., et al, 2024b | 16 | 4 | 100 |
| De Araújo, C. R. B. et al, 2024 | 15 | 5 | 100 |
| Brunacci, K. A. et al, 2024 | 16 | 4 | 100 |
| Bassetti, E. et al, 2023 | 15 | 5 | 100 |
| Santos, M. et al, 2022 | 16 | 4 | 100 |
| Garro-Mellado, L. et al, 2022 | 15 | 5 | 100 |
| Antignani, A. et al, 2022 | 15 | 5 | 100 |
| Alexy, U. et al, 2022 | 15 | 5 | 100 |
| Katiforis, I. et al, 2021 | 15 | 5 | 100 |
| Hutchinson, J. et al, 2021 | 15 | 5 | 100 |
| Grammatikaki, E. et al, 2021 | 15 | 5 | 100 |
| De Araújo, C. R. B. et al, 2021 | 15 | 5 | 100 |
| Theurich, M. A. et al, 2020 | 15 | 5 | 100 |
| Padarath, S. et al, 2020 | 15 | 5 | 100 |
| Pace, L. et al 2020 | 14 | 5 | 100 |
| Moumin, N. A. et al, 2020 | 15 | 5 | 100 |
| Garcia, A. L. et al 2020 | 15 | 4 | 94 |
| Garcia, A. L. et al, 2024 | 16 | 4 | 100 |
| Garcia, A. L. et al, 2022 | 16 | 4 | 100 |
| Simmonds, L. et al, 2021 | 14 | 5 | 93 |
| McCann, J. R., 2020 | 16 | 4 | 100 |
| McCann, J. R., 2021 | 15 | 5 | 100 |
| Dixon, H. G. et al, 2024 | 16 | 4 | 100 |
| Hollinrake, G. et al, 2024 | 16 | 4 | 100 |
| Rowan, M. et al, 2022 | 15 | 4 | 94 |
| McCann, J. et al, 2022 | 17 | 2 | 81 |
| Isaacs, A. et al, 2022 | 16 | 4 | 100 |

† Quality Assessment Carried out Using BMJ Appraisal Tool for Cross-Sectional Studies (BMJ, 2016)
